# Influence of Different Low-Temperature Treatments on Chilling Injury and Accumulation of Characteristic Anthocyanins in Pomegranates

**DOI:** 10.3390/foods14193422

**Published:** 2025-10-04

**Authors:** Pan Shu, Yuan Qing, Jianping Hu, Xin Yao, Jing Li, Lin Shen

**Affiliations:** 1 Sichuan Technological Innovation Laboratory for South Subtropical Fruits, College of Agricultural Science, Xichang University, Xichang 615013, China; xcc20240018@xcc.edu.cn (P.S.); sp199546cau@163.com (J.H.); qyxccedu@163.com (X.Y.); yxxccedu@163.com (J.L.); 2 College of Food Science and Nutritional Engineering, China Agricultural University, Beijing 100083, China

**Keywords:** pomegranate, anthocyanidins, chilling injury, metabolomic

## Abstract

Low-temperature storage causes chilling injury (CI) in pomegranate fruit and influences anthocyanin accumulation. However, the exploration of characteristic anthocyanins in pomegranates and their association with CI remains poorly understood. In this study, the “Acid” variety displayed fewer CI symptoms, a lower rate of weight loss, and higher redness intensity compared to “Soft seeds” and “Six-month red”. Peel anthocyanin content declined during cold storage, with a slower decrease at 10 °C than that at 4 °C. However, storage at 4 °C reduced the aril anthocyanin content in “Six-month red” and “Soft seeds” pomegranates, but promoted its accumulation in “Acid”. At 10 °C, aril anthocyanin in “Six-month red” was unaffected, whereas accumulation was observed in “Soft seeds” and “Acid”. Analysis identified 103 anthocyanins in total, 25 of which were common to both peel and aril. Cyanidin-3,5-O-diglucoside and pelargonidin-3,5-O-diglucoside were present in both the peel and aril of “Six-month red” and “Acid” varieties, with higher contents than in “Soft seeds”. Low temperature affected both characteristic anthocyanins and key synthesis genes (*PgDFR*, *PgUFGT*, *PgANS*, *PgF3′H*, *PgCHI*), with effects consistent with those on total anthocyanins. The “Acid” variety exhibits high cold tolerance, which alleviates peel anthocyanin degradation and promotes aril anthocyanin accumulation. These findings will contribute to elucidating the mechanisms underlying cold tolerance in pomegranates and provide new insights for pomegranate breeding.

## 1. Introduction

Pomegranates (*Punica granatum* L.) are rich in bioactive components, including anthocyanins, flavonoids, punicalagin, and tannins [1,2]. Anthocyanidins, a class of natural water-soluble pigments within the flavonoid family, are synthesized in the cytoplasm and subsequently translocated to the vacuole [3]. They can be commonly found in roots, stems, leaves, flowers, fruits, and other plant organs, functioning as secondary metabolites crucial for plant growth, development, and responses to environmental stresses [4]. Anthocyanidins, being inherently unstable, typically form stable anthocyanins through glycosidic bonding with sugar molecules. Anthocyanins exhibit stability within cellular pH ranges and are stored in high concentrations in vacuoles, contributing to the distinct red, purple, and blue hues observed in plant tissues as a result of varying types and levels of anthocyanin accumulation [5].

Low-temperature storage is a commonly utilized method to prolong the postharvest lifespan of horticultural commodities, preserving their nutraceutical quality [6]. Pomegranates, native to subtropical regions and of high economic value, are particularly susceptible to chilling injury (CI) (4 °C or 5 °C) [7,8]. CI stimulates reactive oxygen species (ROS) to be generated, triggering oxidative harm and peel discoloration in the fruits. Various exogenous compounds, such as arginine, 24-epibrassinolide, and methyl jasmonate, have been proven effective in alleviating cold stress in pomegranates [9,10,11]. Additionally, the impact of low temperature on anthocyanin level varies among different species. In apples, low-temperature signaling induces MdMYB2 binding to the *MdSIZ1* promoter, promoting its transcription and subsequent upregulation of anthocyanin biosynthetic genes, thereby enhancing anthocyanin accumulation [12]. Postharvest storage of plum fruits at low temperatures enhances anthocyanin content in the flesh of black/purple plums, resulting in flesh reddening [13]. Under cold stress, MdMYB308L interacts with MdbHLH33 to synergistically activate *MdDFR*, consequently boosting anthocyanin accumulation [14]. Conversely, in strawberry fruits, low temperatures stimulate the expression of FaSnRK2.6, an *Arabidopsis* ortholog that acts as a negative regulator, inhibiting anthocyanin production and accumulation [15]. Importantly, varying low-temperature conditions yield differing impacts on anthocyanins. Blood oranges stored at 8 °C [16] or 9 °C [17] produce more anthocyanins than those stored at 4 °C. Additionally, anthocyanin accumulation can reduce oxidative damage, increase photosynthetic rates, alleviate growth inhibition, and prevent cell death [18]. Anthocyanins can serve as osmotic regulators, directly enhancing cold tolerance [19]. However, existing studies have not explored the dynamic alteration of anthocyanins in pomegranate fruits under different low-temperature conditions.

The biosynthesis of anthocyanins mainly occurs through the phenylpropanoid pathway [20,21]. Phenylalanine is converted into coumaroyl-CoA, a precursor of the downstream flavonoid pathway, catalyzed by phenylalanine ammonialyase (PAL), cinnamic acid 4-hydroxylase (C4H), and 4-coumarate coenzyme A ligase (4CL). Coumaroyl-CoA is transformed into dihydromyricetin sequentially under the catalysis of chalcone synthase (CHS), chalcone isomerase (CHI), flavanone-3-hydroxylase (F3H), flavonoid 3′-hydroxylase (F3′H), and flavonoid 3′5′-hydroxylase (F3′5′H). Dihydroflavonols, dihydroquercetin, and dihydromyricetin are converted into cyanidin, pelargonidin, and delphinidin by dihydroflavonol 4-reductase (DFR) and anthocyanidin synthase (ANS). At this stage, the colorless anthocyanidin precursors are converted into colored anthocyanidins, which are then glycosylated by flavonoid 3-O-glucosyltransferase (UFGT) to form cyanidin glycosides, pelargonidin glycosides, and delphinidin glycosides. Subsequently, various anthocyanins are generated through modifications such as acylation and methylation.

However, most current studies have concentrated on anthocyanin synthesis regulation, while research and analysis of specific anthocyanin species and contents and characteristic anthocyanins are scarce. Additionally, whether the levels of characteristic anthocyanins dominate the effects of low temperature on anthocyanin levels remains unclear. We hypothesize that variety-specific anthocyanin profiles correlate with CI tolerance. Three major anthocyanin constituents, namely cyanidin 3-O-glucoside, cyanidin 3-O-6″-malonyl-glucoside, and cyanidin O-syringic acid, have been identified in red pericarp [22]. In peaches, anthocyanins mainly consist of cyanidin 3-O-glucoside and cyanidin 3-O-rutinoside, which exhibit good nutritional properties and impart a desirable red color to the flesh [23]. Regarding litchi (*Litchi chinensis*), the primary anthocyanin components in its pericarp encompass cyanidin 3-O-glucoside (92%) and pelargonidin 3-O-glucoside (8%) [24]. Additionally, cyanidin 3-galactoside is the most abundant component in apples and red pears [25,26]. In this study, we aimed to characterize the changes in anthocyanin levels in different tissues of pomegranate fruit under varying low-temperature conditions. We further analyzed the key anthocyanins in pomegranate peels and arils, as well as the effects of low temperature on these compounds, to clarify the relationship between the level of key anthocyanins and chilling injury in pomegranates. These findings scientifically benefit the further breeding of new pomegranate varieties.

## 2. Materials and Methods

### 2.1. Fruit Materials and Treatment

Fruits of three pomegranate varieties, locally named “Six-month red”, “Acid”, and “Soft seeds”, were obtained from a pomegranate orchard in Huili, Sichuan, China, and harvested on 23 September 2024 (135 days after fruit set). During the collection process, nine trees of each of the three varieties were randomly selected, and 30 fruits were randomly chosen from each tree to ensure uniformity in color and size. The varieties were “Soft seeds” (weight: 350–360 g, TSS: 13.9–14.1 °Bx, firmness: 1.3–1.5 kg cm^−1^); “Six-month red” (weight: 330–340 g, TSS: 14.9–15.1 °Bx, firmness: 0.8–1 kg cm^−1^); and “Acid” (weight: 430–440 g, TSS: 13.3–13.5 °Bx, firmness: 1.3–1.5 kg cm^−1^). Immediately after the fruits were picked, they were moved to the laboratory. The study only selected fruits without any mechanical damage that were free from pests and diseases for the determination of physiological indices. Then, 205 homogeneous fruits were selected. From these, 3 replicates of 5 fruits each were used to determine physiological indicators and conduct targeted metabolomics sequencing. The remaining 190 fruits were randomly divided into two lots for cold treatment. A total of 95 fruits were kept at 4 ± 1 °C or 10 ± 1 °C with 90 ± 2% relative humidity for 45 days in an artificial climate illumination incubator (HM-P1200D4, Shandong Hengmei Electronic Technology Co., Ltd., Shanghai, China). Among these, 50 fruits (10 per time point) were used for chilling injury phenotype observation. From the remaining 45 fruits, 3 fruits were sampled at each low-temperature treatment time point (0 d, 7 d, 15 d, 30 d, and 45 d), with three replicates. These samples underwent liquid nitrogen freezing and were preserved at −80 °C for later analysis.

“Six-month red” peel was abbreviated as “Sx-P”, “Acid” peel was abbreviated as “Ai-P”, and “Soft seeds” peel was abbreviated as “St-P”. “Six-month red” aril was abbreviated as “Sx-Ar”, “Acid” aril was abbreviated as “Ai-Ar”, and “Soft seeds” aril was abbreviated as “St-Ar”.

### 2.2. Anthocyanin Extraction and Determination

The pH contrast method was adopted for confirming the anthocyanin content; when pH = 1.0, anthocyanin has a characteristic absorption peak at 530 nm, and when pH = 4.5, anthocyanin transforms into a colorless chalcone form with no absorption peak at 530 nm. The absorbance values at 530 and 700 nm were determined under varying pH conditions, followed by anthocyanin content being quantitatively measured. We accurately weighed 0.2 g of pomegranate tissue, added 1 mL of extraction solution, and fully ground it into a homogenate using a pestle. We extracted this at 60 °C for 30 min (sealed to prevent moisture loss), with shaking and mixing several times during the process. This was followed by 10 min of centrifugation at 12,000 rpm at room temperature (RT), and the supernatant obtained was the sample to be tested. For detailed procedures, refer to Beijing Boxbio Science & Technology Co., Ltd. (AKPL021M kit, Beijing, China).

### 2.3. Weight Loss Rate

The pomegranate fruits of the three varieties were numbered (n = 10), and their initial weights were measured as m0_1–10_. After cold stress treatment on different days, the fruits were placed at RT for three days, and their weights were measured as m1_1–10_. The CI symptoms of fruits usually require 3 days of storage at RT to fully manifest, and the weights of the fruits change accordingly during this period. In a low-temperature environment the humidity is controlled, which cannot properly reflect the weight change in fruits during shelf life. Therefore, we chose to measure weight loss as following when the CI phenotype is fully expressed:

Weight loss rate (%) = [(m0_1–10_ − m1_1–10_)/m0_1–10_] × 100

### 2.4. Chilling Injury (CI) Measurement

CI was evaluated by visually assessing husk browning on 10 fruits using a hedonic scale from 0 to 3, where 0 indicated no browning, 1 (1–25%), 2 (26–50%), and 3 (>51%), using the following formula:

CI index = [∑ (scale value × number of fruits at that value)]/(total number of fruits × 4)

### 2.5. Color Change

Fruit color was determined as described in our previous study [27]. Fruit color was measured at two symmetrical equatorial points using a Minolta CR-200 colorimeter (Minolta Co., Ltd., Osaka, Japan). The a* value was recorded to represent the red–green axis, with positive and negative values corresponding to red and green coloration, respectively.

### 2.6. Gene Expression Analysis

A TransZol Up Plus RNA kit (TransGen Biotech, Beijing, China) was adopted for isolating total RNA from pomegranate fruit, followed by the elimination of genomic DNA and the synthesis of cDNA via a TransScript One-step gDNA removal and a cDNA Synthesis SuperMix (TransGen Biotech). We performed quantitative real-time PCR (RT-qPCR) on a QuantStudio 1 Plus with Hiscript III RT SuperMix for qPCR (Vazyme, Nanjing, China). Primers targeting coding sequences (CDSs) were designed using SnapGene software 5.3 (Table S1). The 2^−ΔΔCT^ method was adopted for the calculation of the relative gene expression level, with *actin7-like* (LOC116200207) serving as the internal reference gene for data normalization [28].

### 2.7. Targeted Metabolomics Sequencing Analysis

Wuhan Metware Biotechnology (Wuhan, China) performed targeted metabolite profiling by virtue of ultra-performance liquid chromatography (UPLC; ExionLC™ AD, SCIEX, Framingham, MA, USA) alongside tandem mass spectrometry (MS/MS; QTRAP^®^ 6500+, SCIEX). Freeze-dried samples were ground to a powder (30 Hz, 1.5 min) and preserved at −80 °C. For extraction, 50 mg of powder was homogenized with 0.5 mL methanol/water/hydrochloric acid (500:500:1, *v*/*v*/*v*) and vortexed (5 min). After 5 min of sonication, powder samples underwent 3 min of centrifugation at 12,000 g at 4 °C. The residue was then subjected to a second identical extraction. Combined supernatants were filtered (0.22 μm membrane) prior to LC-MS/MS analysis. A UPLC-ESI-MS/MS system was employed for analyzing the sample extracts. The analytical conditions: The UPLC employed an Agilent SB-C18 column (1.8 µm, 2.1 × 100 mm). The mobile phase encompassed solvent A (pure water with 0.1% formic acid) and solvent B (acetonitrile with 0.1% formic acid). The effluent was directed into an ESI-triple quadrupole-linear ion trap (QTRAP)-MS. Metabolites were identified using databases and quantitatively analyzed based on standard substances. For two-group analysis, VIP (VIP > 1) and absolute Log_2_FC (|Log_2_FC| ≥ 1.0) were taken as thresholds to confirm differential metabolites. FDR correction was used for multiple tests. We extracted VIP values from OPLS-DA results, which also contained score plots and permutation plots (R package MetaboAnalystR 4.0). The data was log transformed (log_2_) and mean centered before OPLS-DA. We performed a permutation test (200 permutations) for the avoidance of overfitting.

### 2.8. Statistical Analysis

The entire experiment was repeated 3 times. The data are in the format of means ± standard deviations (SDs). Assessment of statistical difference was conducted by t-test or one-way ANOVA (the SPSS 22.0 software, IBM, NY) alongside Duncan’s multiple range test at ns *p* > 0.05, * *p* < 0.05, ** *p* < 0.01, *** *p* < 0.001, and **** *p* < 0.0001. All figures were generated by GraphPad Prism 8.

## 3. Results

### 3.1. The Effect of Low Temperature on Chilling Injury and Anthocyanin Content in Pomegranates

**Figure 1:**
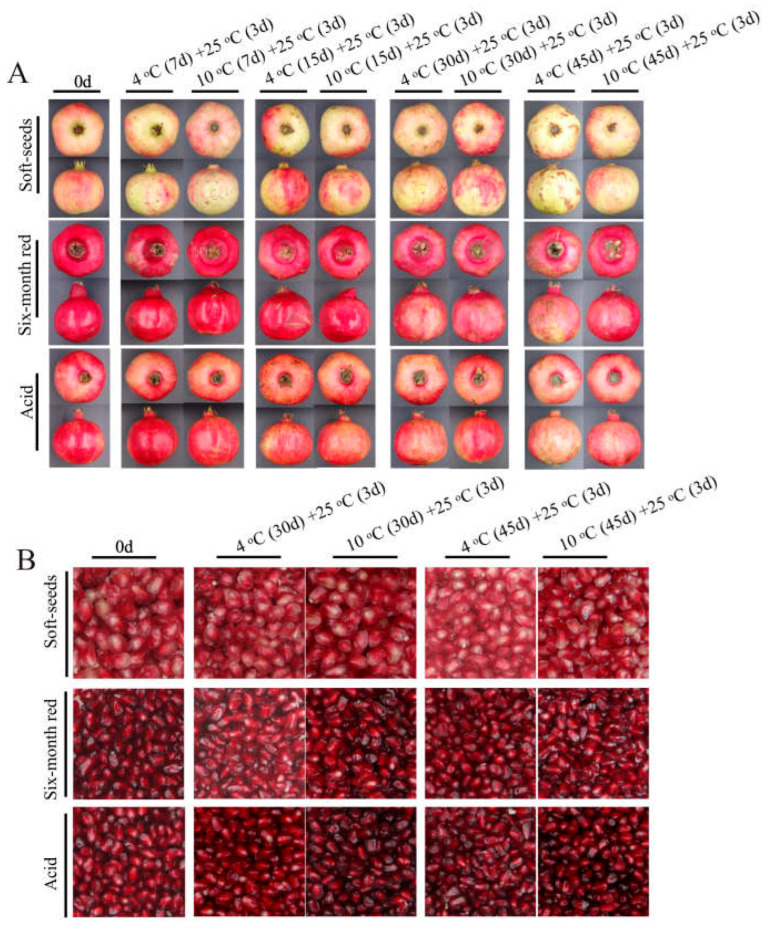
Phenotypic images of “Acid” “Soft seeds” and “Six-month red” after treatment at low temperatures of 4 °C and 10 °C for 7 d, 15 d, 30 d, and 45 d followed by storage at 25 °C for 3 d (**A**). Aril images of “Acid”, “Soft seeds” and “Six-month red” stored at 4 °C and 10 °C for 30 days and 45 days followed by storage at 25 °C for 3 days (**B**).

**Figure 2:**
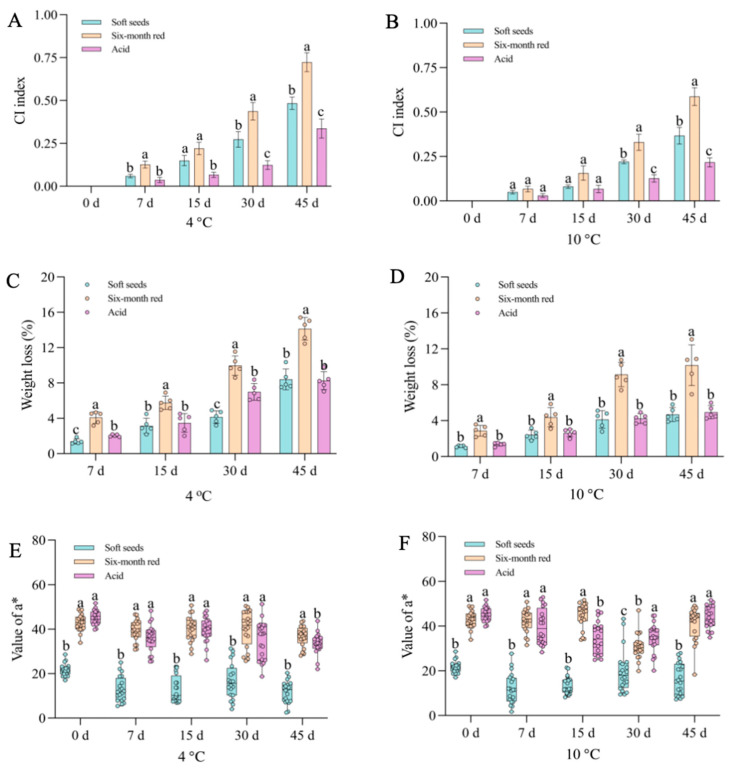
Chilling injury (CI) indexes (**A**,**B**), weight loss rates (**C**,**D**), and color difference a* values (**E**,**F**) of three pomegranate varieties (Soft seeds, Six-month red, and Acid) during different storage periods at 4 °C and 10 °C. All data are presented as the mean ± SD (n = 3). Significantly different values are denoted by distinct letters.

**Figure 3:**
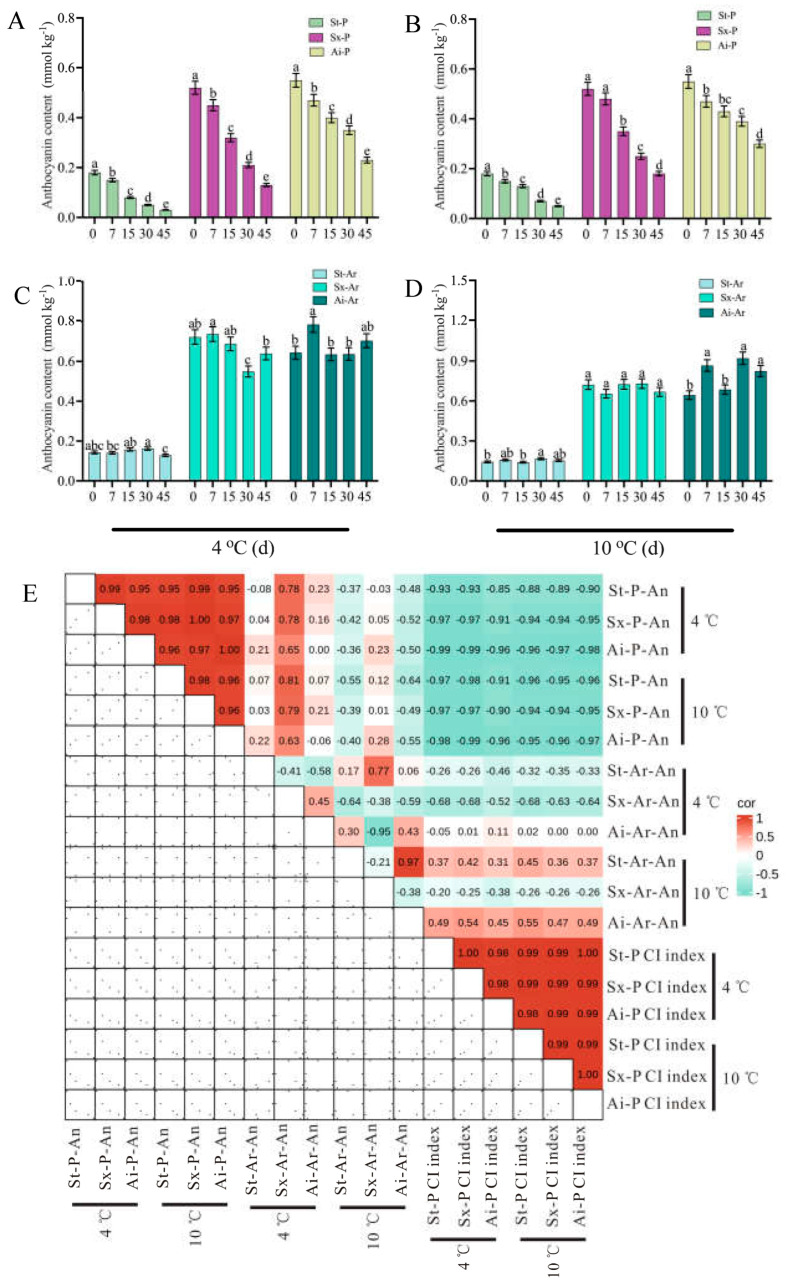
Changes in anthocyanin content in the peels and arils of “Acid”, “Soft seeds”, and “Six-month red” during storage at low temperatures of 4 °C (**A**,**B**) and 10 °C (**C**,**D**) for 0 d, 7 d, 15 d, 30 d, and 45 d. (**E**) Correlation heatmap between peel anthocyanin contents, aril anthocyanin contents of “Acid”, “Soft seeds”, and “Six-month red”, and chilling injury (CI) index. Red represents positive correlation, and cyan represents negative correlation. Anthocyanin was abbreviated as “An”. The peels of “Six-month red”, “Acid”, and “Soft seeds” were abbreviated as “Sx-P”, “Ai-P”, and “St-P”, respectively. The arils of “Six-month red”, “Acid”, and “Soft seeds” were abbreviated as “Sx-Ar”, “Ai-Ar”, and “St-Ar”, respectively. All data are presented as the mean ± SD (n = 3). Different letters denote significant differences.

Three pomegranate varieties exhibiting distinct color traits were selected for a cold stress study. The findings demonstrated that prolonged exposure to low temperatures induced evident CI symptoms in pomegranates, with severity escalating as temperature decreased (Figure 1A). Additionally, the coloration of the arils was impacted by low temperature, with varying degrees of influence observed among the different varieties (Figure 1B). Notably, the “Acid” pomegranate displayed milder CI symptoms compared to the “Soft seeds” and “Six-month red” varieties, with the latter exhibiting the most severe CI symptoms (Figure 1A). Meanwhile, the CI index and weight loss rate were higher in “Six-month red” and “Soft seeds” pomegranates than in “Acid”, which were accompanied by lower a* values (Figure 2A–F). To verify the observed effects of low temperature on peel and aril coloration, the anthocyanin content of these tissues was further determined. The results revealed a progressive decline in peel anthocyanin content with prolonged low-temperature storage across all examined pomegranate varieties, irrespective of their pigmentation intensity (e.g., “Soft seeds” and darker cultivars such as “Six-month red” and “Acid”). This decline was more rapid at lower temperatures (Figure 3A,C). Sx-Ar anthocyanin content declined significantly after 30 days of storage at 4 °C and after 45 days in St-Ar, whereas it increased notably on days 7 and 45 in Ai-Ar. Storage at 10 °C had minimal impact on anthocyanin in Sx-Ar but promoted accumulation in St-Ar and Ai-Ar (Figure 3B,D). Correlation analysis unveiled a positive relationship among the CI index of the diverse pomegranate varieties, alongside a noteworthy negative correlation between the CI index and peel anthocyanin content (Figure 3E), indicating a close association between anthocyanin levels and fruit CI.

### 3.2. Metabolomics Reveals Variation in Anthocyanin Numbers in Different Pomegranate Varieties

**Figure 4:**
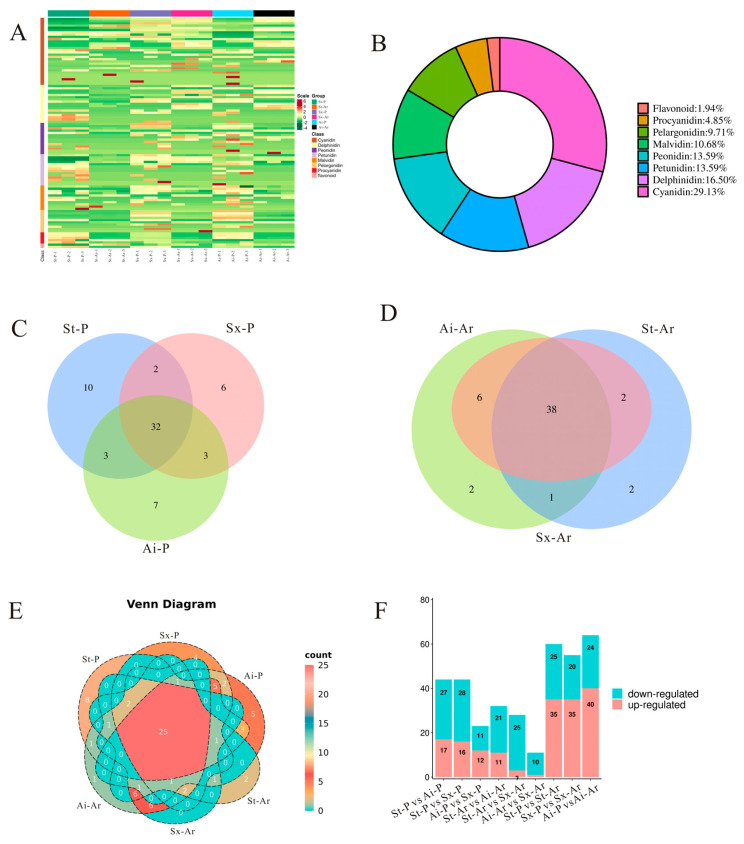
Analysis of anthocyanin contents and types in pomegranate peels and arils. Heatmap analysis of anthocyanins detected in the peel and aril of three pomegranates (**A**); donut chart illustrating the specific categories and proportional distribution of detected anthocyanins (**B**); Venn diagram analysis of anthocyanin intersections among St-P, Ai-P, and Sx-P (**C**); Venn diagram analysis of anthocyanin intersections among St-Ar, Ai-Ar, and Sx-Ar (**D**); Venn diagram analysis of the number of anthocyanins shared between pomegranate peels and arils (**E**); quantitative comparison of differentially accumulated anthocyanins in pairwise analyses of peel and aril tissues among three pomegranate cultivars (**F**). The peels of “Six-month red”, “Acid”, and “Soft seeds” were abbreviated as “Sx-P”, “Ai-P”, and “St-P”, respectively. The arils of “Six-month red”, “Acid” and “Soft seeds” were abbreviated as “Sx-Ar”, “Ai-Ar”, and “St-Ar”, respectively.

In our study aimed at identifying the specific anthocyanins responsible for pigmentation in pomegranate peels and arils, targeted metabolomics were conducted to quantitatively compare anthocyanin profiles among three representative varieties. A heatmap was created to visualize the relative contents of 103 anthocyanins (Figure 4A). Additionally, the analysis showed that these anthocyanins primarily belonged to the eight categories, with cyanidin comprising the highest proportion (29.13%), followed by delphinidin (16.5%), petunidin (13.59%), and peonidin (13.59%) (Figure 4B). Further analysis revealed that 47, 43, and 45 anthocyanins were detected in St-P, Sx-P, and Ai-P, respectively, with 32 anthocyanins being commonly shared among all three varieties (Figure 4C). In the arils, 43, 46, and 47 anthocyanins were identified in St-Ar, Sx-Ar, and Ai-Ar, respectively, among which 38 anthocyanins were consistently detected across all three varieties (Figure 4D). Notably, 25 anthocyanins were shared between peels and arils (Figure 4E). Analysis of differential anthocyanin levels revealed that the light-colored “Soft seeds” exhibited a greater number of downregulated anthocyanins compared to upregulated anthocyanins when compared to the dark-colored “Six-month red” and “Acid” pomegranates. Furthermore, comparative analysis within the same variety indicated that arils had more upregulated anthocyanins compared with peels (Figure 4F).

### 3.3. Analysis of the Characteristic Anthocyanins of the Pomegranate Peels

**Figure 5:**
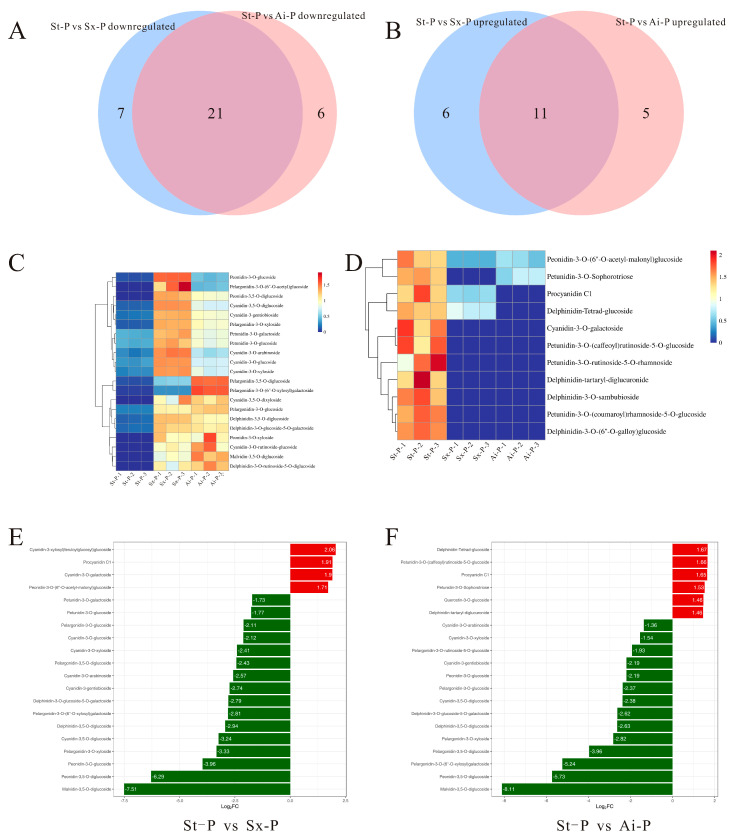
Venn diagrams were used to analyze the intersections of upregulated anthocyanins between the St-P and Sx-P, as well as between St-P and Ai-P, and the intersections of downregulated anthocyanins in the same comparison groups (**A**,**B**); heatmap analysis of anthocyanins that are commonly upregulated and downregulated in the peel (**C**,**D**); the top 20 anthocyanins with differential fold-changes in St-P vs. Sx-P and St-P vs. Ai-P (**E**,**F**). The peels of “Six-month red”, “Acid”, and “Soft seeds” were abbreviated as “Sx-P”, “Ai-P”, and “St-P”, respectively.

Although the number of detected anthocyanins did not exhibit significant variation among the different pomegranate varieties, these findings suggest that fruit color is predominantly influenced by the levels of key anthocyanin constituents. To elucidate the specific anthocyanins associated with the decrease induced by low temperatures, we characterized characteristic anthocyanins in the peels and arils of diverse pomegranate varieties. Venn diagrams depict the intersections of upregulated anthocyanins between the St-P and Sx-P, as well as between St-P and Ai-P, along with the overlap of downregulated anthocyanins within the same comparison groups (Figure 5A,B).

**Figure 6:**
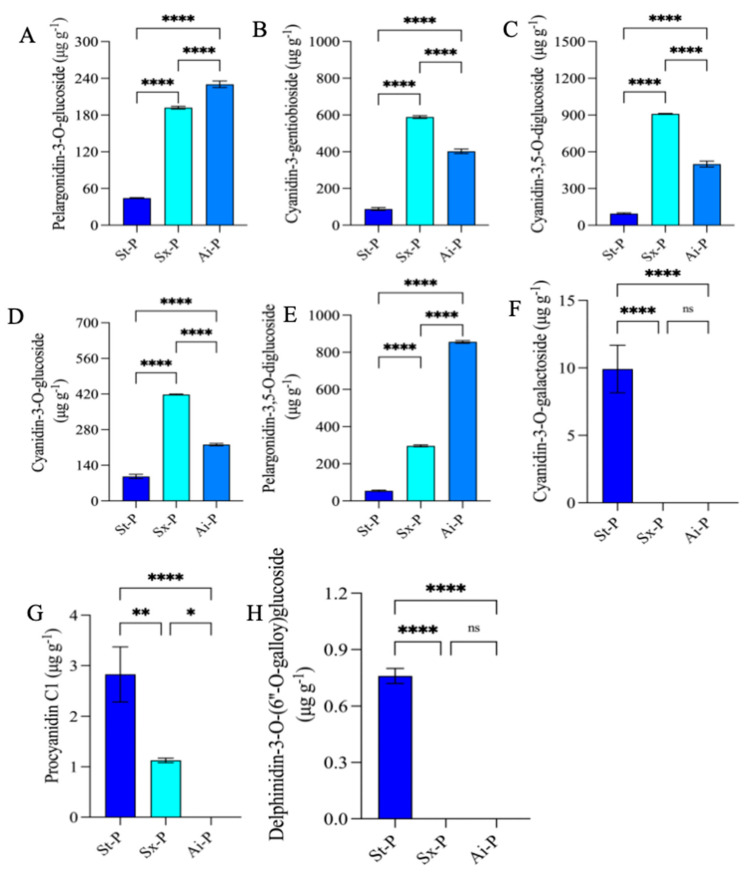
Contents of pelargonidin-3-O-glucoside, cyanidin-3-O-gentiobioside, cyanidin-3,5-O-diglucoside, cyanidin-3-O-glucoside, pelargonidin-3,5-O-diglucoside, cyanidin-3-O-galactoside, procyanidin C1, and delphinidin-3-O-(6”-O-galloyl)glucoside in St-P, Sx-P, and Ai-P (**A**–**H**). All data are represented by the mean ± SD (n = 3). ns *p* > 0.05, * *p* < 0.05, ** *p* < 0.01, **** *p* < 0.0001. The peels of “Six-month red”, “Acid”, and “Soft seeds” were abbreviated as “Sx-P”, “Ai-P”, and “St-P”, respectively.

The analysis identified 21 commonly upregulated anthocyanins at the intersections, and their expression levels were depicted through a heatmap (Figure 5C). By combining these 21 shared anthocyanins with the top 20 fold-change anthocyanins, it was determined that pelargonidin-3-O-glucoside, cyanidin-3-O-gentiobioside, cyanidin-3,5-O-diglucoside, cyanidin-3-O-glucoside, and pelargonidin-3,5-O-diglucoside exhibited the highest contents meeting the top 20 fold-change criteria (Figure 5E). Additionally, their contents were greatly lower than those in Sx-P and Ai-P (Figure 6A–E). Furthermore, 11 common downregulated anthocyanins were identified in the intersections, and their expression levels were also illustrated using a heatmap (Figure 5D).

According to a combined analysis of these 11 shared anthocyanins with the top 20 fold-change anthocyanins, it was revealed that cyanidin-3-O-galactoside, procyanidin C1, and delphinidin-3-O-(6”-O-galloy)glucoside had the highest contents meeting the top 20 criteria (Figure 5F). Conversely, their levels were remarkably lower in St-P versus Sx-P and Ai-P (Figure 6F–H).

### 3.4. Analysis of the Characteristic Anthocyanins of the Pomegranate Arils

**Figure 7:**
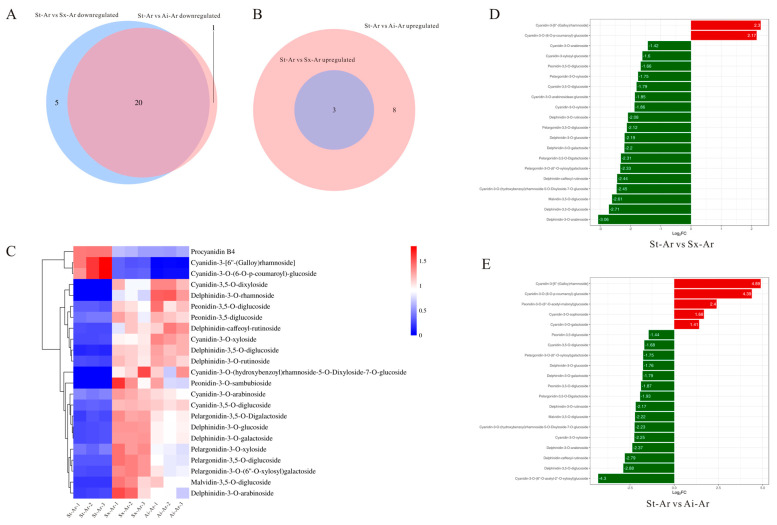
Venn diagrams were used to analyze the intersections of upregulated anthocyanins between St-Ar and Sx-Ar, as well as between St-Ar and Ai-Ar, and the intersections of downregulated anthocyanins in the same comparison groups (**A**,**B**); heatmap analysis of commonly upregulated and downregulated anthocyanins in the aril (**C**); top 20 anthocyanins with the highest fold-changes in St-Ar vs. Sx-Ar and St-Ar vs. Ai-Ar (**D**,**E**). The arils of “Six-month red”, “Acid”, and “Soft seeds” were abbreviated as “Sx-Ar”, “Ai-Ar”, and “St-Ar”, respectively.

**Figure 8:**
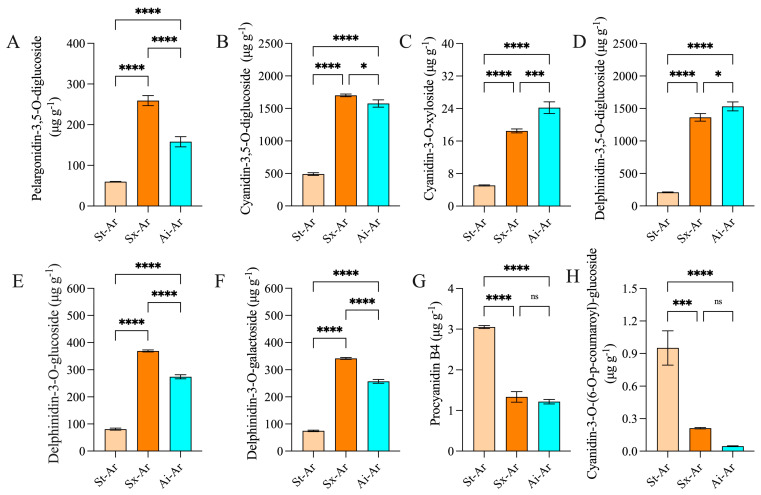
Contents of pelargonidin-3,5-O-diglucoside, cyanidin-3,5-O-diglucoside, cyanidin-3-O-xyloside, delphinidin-3,5-O-diglucoside, delphinidin-3-O-glucoside, delphinidin-3-O-galactoside, procyanidin B4, and cyanidin-3-O-(6-O-p-coumaroyl)-glucoside in St-Ar, Sx-Ar, and Ai-Ar (**A**–**H**). All data represent the mean ± SD (n = 3). ns *p* > 0.05, * *p* < 0.05, *** *p* < 0.001, **** *p* < 0.0001. The arils of “Six-month red”, “Acid”, and “Soft seeds” are expressed as “Sx-Ar”, “Ai-Ar”, and “St-Ar”, respectively.

We further analyzed the characteristic anthocyanins in the arils of different pomegranate fruits. Venn diagram results revealed 20 common intersections of downregulated anthocyanins between St-Ar vs. Sx-Ar and St-Ar vs. Ai-Ar, with only four specific anthocyanins present (Figure 7A). This suggests that the anthocyanin levels in arils may be closely related to the contents of specific anthocyanins rather than special ones. Additionally, three commonly upregulated anthocyanins were found in arils (Figure 7B). We used a heatmap to visualize the contents of these 23 common anthocyanins in different pomegranate fruits (Figure 7C). To screen the main representative anthocyanin components, we further conducted a joint analysis of these 23 common anthocyanins and the top 20 anthocyanins with the highest fold-changes. The results showed that pelargonidin-3,5-O-diglucoside, cyanidin-3,5-O-diglucoside, cyanidin-3-O-xyloside, dephinidin-3,5-O-diglucoside, delphinidin-3-glucoside, delphinidin-3-O-galactoside, procyanidin B4, and cyanidin-3-O-(6-)-p-coumaroyl)-glucoside had the highest contents or fold-changes meeting the top 20 criteria (Figure 7D,E). Furthermore, the contents of these anthocyanins in St-Ar are dramatically lower than those in Sx-Ar and Ai-Ar, except for procyanidin B4 and cyanidin-3-O-(6-O-p-coumaroyl)-glucoside (Figure 8A–H).

### 3.5. Effect of Low Temperature on the Contents of Characteristic Anthocyanins in Arils and Anthocyanin Synthesis Genes

**Figure 9:**
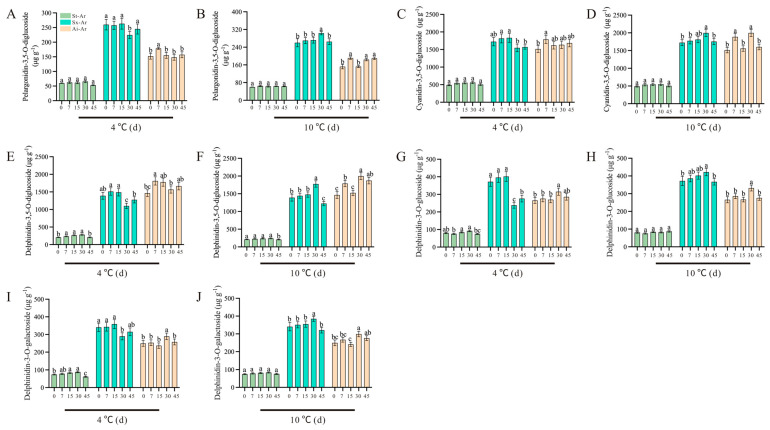
Contents of pelargonidin-3,5-O-diglucoside, cyanidin-3,5-O-diglucoside, delphinidin-3,5-O-diglucoside, delphinidin-3-O-glucoside, and delphinidin-3-O-galactoside in St-Ar, Sx-Ar, and Ai-Ar under treatments at 4 °C and 10 °C, respectively (**A**–**J**). All data represent the mean ± SD (n = 3). Different letters represent significant differences. The arils of “Six-month red”, “Acid”, and “Soft seeds” were abbreviated as “Sx-Ar”, “Ai-Ar”, and “St-Ar”, respectively.

We conducted a targeted metabolomics analysis to compare and evaluate the specific anthocyanin contents in pomegranate varieties with distinct colors. Subsequently, we identified five characteristic anthocyanins with notable contents and significant fold-changes in the aril. However, it remained unclear whether these specific anthocyanins were influenced by low temperatures and how their levels varied under different temperature conditions. To address this, we examined the alterations in the content of these five characteristic anthocyanins under low temperatures (4 °C and 10 °C). Our results indicated that, pelargonidin-3,5-O-diglucoside and cyanidin-3,5-O-diglucoside in St-Ar were not significantly affected by low temperature (Figure 9A–D).

**Figure 10:**
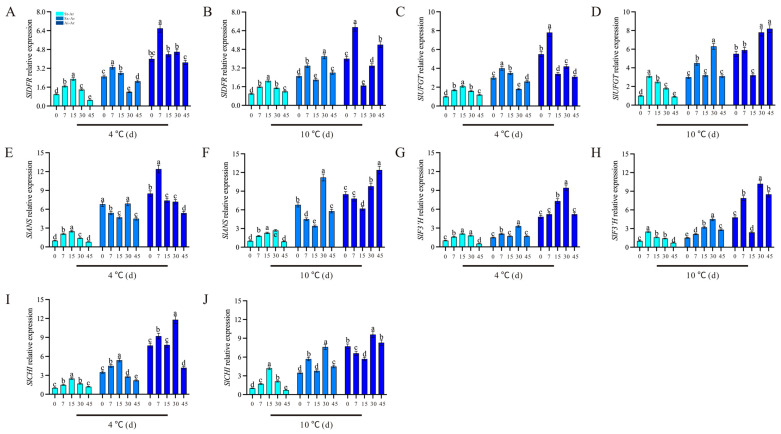
Expression levels of *PgDFR*, *PgUFGT*, *PgANS*, *PgF3′H*, and *PgCHI* in St-Ar, Sx-Ar, and Ai-Ar under treatments at 4 °C and 10 °C, respectively (**A**–**J**). All data represent the mean ± SD (n = 3). Different letters stand for significant differences. The arils of “Six-month red”, “Acid”, and “Soft seeds” were abbreviated as “Sx-Ar”, “Ai-Ar”, and “St-Ar”, respectively.

Conversely, the contents of pelargonidin-3,5-O-diglucoside and cyanidin-3,5-diglucoside in Sx-Ar notably decreased after a 30-day treatment at 4 °C but significantly increased following a 30-day treatment at 10 °C (Figure 9B). Similarly, the contents of pelargonidin-3,5-O-diglucoside and cyanidin-3,5-O-diglucoside contents in Ai-Ar also significantly increased after a 30-day treatment at 10 °C (Figure 9B,D). Additionally, we observed similar trends in the changes in dephinidin-3,5-o-diglucoside, delphinidin-3-o-glucoside, and delphinidin-3-o-galactoside, which all exhibited significant increases after a 30-day treatment at 10 °C (Figure 9E–J). Furthermore, we measured the expression levels of key genes in the synthesis pathways of pelargonidin, cyanidin, and delphinidin. Our findings revealed that the expression patterns of *PgDFR*, *PgUFGT*, *PgANS*, *PgF3′H*, and *PgCHI* under low temperatures were similar to those of the above five characteristic anthocyanins. Specifically, these genes demonstrated a decrease after a 30-day treatment at 4 °C and an increase following a 30-day treatment at 10 °C (Figure 10A–J).

## 4. Discussion

Anthocyanins are key pigments influencing fruit coloration, with their accumulation being influenced by environmental factors such as low temperature [29,30,31]. Notably, different low temperatures exert distinct effects on anthocyanin accumulation. For instance, in blood oranges, anthocyanin accumulation is greater at 9 °C than at 4 °C [17]. Our study revealed that anthocyanin accumulation is not only impacted by temperature but also by tissue type and cultivar. Specifically, we revealed a progressively decreased peel anthocyanin content during low-temperature treatment, with a slower decline rate at 10 °C than at 4 °C. In contrast, the aril anthocyanin content of the “Acid” pomegranate accumulated significantly at both 4 °C and 10 °C. However, for the “Soft seeds” and “Six-month red” pomegranates—cultivars with poor cold resistance—the aril anthocyanin content decreased after 45 days of storage at 4 °C, while no significant change was observed at 10 °C. This observation suggests a close association between this phenomenon and the inherent low-temperature tolerance of the fruit. Therefore, we analyzed the correlation between anthocyanin content and chilling injury symptoms, revealing a significant negative correlation between them. In plant species rich in anthocyanins, tissue tolerance to low-temperature stress can be achieved without the need for additional anthocyanin synthesis. Conversely, in plants lacking anthocyanins, exposure to low temperature stress triggers anthocyanin biosynthesis by activating transcription factors and structural genes. Notably, stress tolerance is positively correlated with anthocyanin content [32]. Studies conducted on strawberries and apples have demonstrated that anthocyanin accumulation enhances resistance to cold stress [33,34].

Anthocyanins are primarily categorized into six major categories: pelargonidin, malvidin, peonidin, petunidin, delphinidin, and cyanidin. However, comprehensive research on characteristic anthocyanins in different fruit parts remains limited. Furthermore, dynamic analyses of these characteristic anthocyanins under low-temperature conditions require further investigation. In this study, we identified 103 distinct anthocyanidins in pomegranate fruits, with derivatives of cyanidin (29.13%), delphinidin (16.5%), and peonidin (13.59%) being the most abundant. This finding aligns with previous reports indicating that approximately 90% of anthocyanins are derived from methylated derivatives of cyanidin, delphinidin, and pelargonidin [35]. Our results are consistent with this. Specifically, cyanidin, the most prevalent anthocyanin, can be found in over 82% of the fruits and berries examined [36]. Metabolomic profiling helped confirm 60 anthocyanin-related metabolites, where cyanidin-3-O-rutinoside was the primary pigment in CL-V-DW [31]. Notably, we observed the highest levels of cyanidin in three different pomegranate species, suggesting a prominent role for cyanidin in fruit coloration. Peel color represents a key quality determinant for market acceptance, with anthocyanin-induced vibrant red hues enhancing aesthetic appeal to consumers [37,38]. Studies have yielded diverse findings regarding anthocyanin composition and concentration in fruit peels. For instance, Tunisian pomegranate peels have been characterized by anthocyanins such as cyanidin and pelargonidin monosubstituted derivatives, including pelargonidin-3-pentoside, cyanidin-3-glucoside, rutinoside, and pentoside derivatives [39]. We found that pelargonidin-3-O-glucoside, cyanidin-3-O-gentiobioside, cyanidin-3,5-O-diglucoside, cyanidin-3-O-glucoside, and pelargonidin-3,5-O-digluco-side had the highest contents and met the top 20 fold-change criteria. Additionally, their contents in St-P were considerably lower than those in Sx-P and Ai-P. Previous research on three distinct Chinese pomegranate cultivars has revealed the variability of six major anthocyanins in fruit peels. Specifically, in red and green cultivars cyanidin-3-glucoside predominated, whereas in deep-red cultivars both cyanidin-3-glucoside and delphinidin-3-glucoside were the predominant constituents [40]. Cyanidin-3,5-O-diglucoside and pelargonidin-3,5-O-diglucoside presented in both the peels and arils of “Six-month red” and “Acid” pomegranates, with higher concentrations observed compared to the ‘Soft-seeds’ variety. Among the seven cultivars in South Africa, cyanidin-3,5-O-diglucoside and delphinidin-3,5-O-diglucoside were identified in “Arakta”, “Bhagwa”, and “Herskawitz”. In contrast, only cyanidin-3,5-O-diglucoside was observed in the “Ganesh”, “Ruby”, and “Wonderful” cultivars [41]. Cyanidin-derived anthocyanins, including cyanidin 3-O-(6′’-malonylglucoside), cyanidin 3-O-glucoside, and cyanidin O-syringic acid, were detected exclusively in red pericarp (RP) longan [22]. These results indicate that while the major types of anthocyanidins are conserved across species and cultivars, their specific contents and glycosylation patterns vary considerably.

The influence of low temperature on anthocyanin accumulation is well-documented. Moderate low temperatures are generally thought to promote the accumulation of anthocyanins through inducing the expression of anthocyanin synthesis-related genes, whereas excessively high or low temperatures can inhibit this process. For instance, in strawberries, increased anthocyanin content has been attributed to the modulating mechanism of *MYB10* and *MYB1* against the expression of structural genes of *ANS* and *UFGT* [42]. Similarly, in blood oranges, low temperature promotes anthocyanin accumulation by activating key synthesis genes, including *ANS*, *CHS*, and *DFR* [43]. In *Broussonetia papyrifera* (paper mulberry), low temperature upregulates *CHS2* expression, promoting anthocyanin synthesis and enhancing cold tolerance [44]. The study focused on measuring the expression levels of anthocyanin synthesis genes at different temperatures and observed that the expression patterns of *PgDFR*, *PgUFGT*, *PgANS*, *PgF3′H*, and *PgCHI* aligned with the trends of characteristic anthocyanins. This confirms that the effect of low temperature on characteristic anthocyanins is closely linked to the activity of anthocyanin synthesis genes. These findings indicate that low temperature-induced changes in characteristic anthocyanin levels are strongly associated with anthocyanin synthesis-genes’ expression.

## 5. Conclusions

Cyanidin-3,5-O-diglucoside and pelargonidin-3,5-O-diglucoside were present in both the peel and aril of the “Six-month red” and “Acid” varieties, with higher contents than those in the “Soft seeds” variety. Low temperature regulates anthocyanin accumulation or degradation in pomegranates by modulating key biosynthetic genes and specific anthocyanin levels, but these effects vary significantly by fruit variety and tissue. “Acid” pomegranates uniquely alleviate peel anthocyanin degradation and promote aril anthocyanin accumulation, thus demonstrating enhanced cold resistance.

## Data Availability

The original contributions presented in the study are included in the article, further inquiries can be directed to the corresponding author.

## Supplementary Materials

The following supporting information can be downloaded at: https://www.mdpi.com/article/10.3390/foods14193422/s1, Table S1: The Primer Sequence for Detecting Gene Expression.

## Abbreviations

The following abbreviations are used in this manuscript:

CI	chilling injury
ROS	reactive oxygen species
PAL	phenylalanine ammonialyase
C4H	cinnamic acid 4-hydroxylase
4CL	4-coumarate coenzyme A ligase
CHS	chalcone synthase
CHI	chalcone isomerase
F3H	flavanone-3-hydroxylase
F3′H	flavonoid 3′-hydroxylase
F3′5′H	flavonoid 3′5′-hydroxylase
DFR	dihydroflavonol 4-reductase
ANS	anthocyanidin synthase
UFGT	flavonoid 3-O-glucosyltransferase
Sx-P	“Six-month red” peel
Ai-P	“Acid” peel
St-P	“Soft-seeds” peel
Sx-Ar	“Six-month red” Aril
Ai-Ar	“Acid” Aril
St-Ar	“Soft-seeds” Aril
RT-qPCR	quantitative real-time PCR
