# Supplementary material for: Influence of Different Low-Temperature Treatments on Chilling Injury and Accumulation of Characteristic Anthocyanins in Pomegranates
Source: Foods. 2025 Oct 4;14(19):3422. doi: 10.3390/foods14193422 (PMC12523530; doi:10.3390/foods14193422)
Supplement: Supplementary file 1 [file foods-14-03422-s001.zip › foods-3845572-supplementary.pdf]

SUPPLEMENTARY INFORMATION

Table S1. The Primer Sequence for Detecting Gene Expression.

Gene	Primer sequence
actin7-like	F: 5'-CCTTGCTGGCCGTGATCTTA-3'; R: 5'-CCAGCTCCTGCTCATAGTCG-3'
PgDFR	F: 5'-TTCAACCCGTCCAGAGACCT- 3' R: 5'-CGGAGGGAAAGAAGGCATGA- 3'
PgUFGT	F: 5'-GCATCGGGCCTTTGAACCTG-3' R: 5'-TTCTGATTGTCGAGCCACGG-3'
PgANS	F: 5'-TGGCCTAAGATCCCGAGTGA-3' R: 5'-AACTTCCTTCTCCAGCCTGC-3'
PgF3'H	F: 5'-ACGGATACCACATCGAGCAC-3' R: 5'-TCGGTCACTAGACGGTCCTT-3'
PgCHI	F: 5'-CACCAAGTGGAAGGGCAAGA-3' R: 5'-CGATTCCTCTTTCCCCTCGG-3'
